# A Primer on Hyperdimensional Computing for iEEG Seizure Detection

**DOI:** 10.3389/fneur.2021.701791

**Published:** 2021-07-20

**Authors:** Kaspar A. Schindler, Abbas Rahimi

**Affiliations:** ^1^Department of Neurology, Inselspital, Sleep-Wake-Epilepsy-Center, NeuroTec, Bern University Hospital, University Bern, Bern, Switzerland; ^2^IBM Research-Zurich, Ruschlikon, Switzerland

**Keywords:** brain-inspired computing, intracranial EEG, epilepsy, hyperdimensional space, digital biomarker, personalized medicine

## Abstract

A central challenge in today's care of epilepsy patients is that the disease dynamics are severely under-sampled in the currently typical setting with appointment-based clinical and electroencephalographic examinations. Implantable devices to monitor electrical brain signals and to detect epileptic seizures may significantly improve this situation and may inform personalized treatment on an unprecedented scale. These implantable devices should be optimized for energy efficiency and compact design. Energy efficiency will ease their maintenance by reducing the time of recharging, or by increasing the lifetime of their batteries. Biological nervous systems use an extremely small amount of energy for information processing. In recent years, a number of methods, often collectively referred to as brain-inspired computing, have also been developed to improve computation in non-biological hardware. Here, we give an overview of one of these methods, which has in particular been inspired by the very size of brains' circuits and termed hyperdimensional computing. Using a tutorial style, we set out to explain the key concepts of hyperdimensional computing including very high-dimensional binary vectors, the operations used to combine and manipulate these vectors, and the crucial characteristics of the mathematical space they inhabit. We then demonstrate step-by-step how hyperdimensional computing can be used to detect epileptic seizures from intracranial electroencephalogram (EEG) recordings with high energy efficiency, high specificity, and high sensitivity. We conclude by describing potential future clinical applications of hyperdimensional computing for the analysis of EEG and non-EEG digital biomarkers.

## 1. Introduction

At the Sleep-Wake-Epilepsy-Center of the University of Bern, we typically see patients who are not seizure free every 3–6 months. These check-ups often also include recording an electroencephalogram (EEG) with extracranial electrodes for a duration of <1 h. This rate of appointments may be considered typical for a tertiary or quaternary epilepsy center in many parts of the world. However, there is huge potential for improvements for several fundamental reasons. It has, for example, been clearly demonstrated that the patients' accounts of seizure occurrences are not reliable (1). The main explanation is not the occasional patient who does not want to report seizures to avoid a suspension of the driver's license or other social and professional consequences, but the fact that many patients are actually willing but not able to give an accurate seizure count. Their seizures may occur during sleep, they may lose consciousness during the seizures, and nobody may tell them afterwards that a seizure occurred, or their seizures may impair their memory, as is typically the case in temporal lobe epilepsy. Furthermore, recent landmark studies (2, 3) have proven that epileptiform activity is far from constant, but exhibits fluctuating dynamics with often robust and patient-specific circadian and multidien periodicities, which are severely under-sampled by the typical sporadic appointment-based short-term EEG recordings (4, 5). Devices that could provide more accurate seizure counts and monitor the individual dynamics of epileptiform activity therefore have a large potential to improve and also personalize the care of epilepsy patients. Nonetheless, a decisive aspect of these devices is that they have to be as unobtrusive and non-visible to others as possible to not aggravate the stigmatization that epilepsy patients are often still exposed to—as most impressively described in the recent autobiography by the American author and journalist Kurt Eichenwald (6). There are several, not mutually exclusive, ways to minimize the obtrusiveness of the devices: one might, for example, restrict EEG recordings to night-time (7), or the device might be attached or integrated into personal accessories. Lee et al. (8) developed a strategy to use elastomeric composites with conductive nanomaterials for designing customized, multifunctional electronic eyeglasses that allow for recording EEG, electrooculogram, ultraviolet intensity, and body movements. Steady skin contact of their highly conductive and deformable carbon nanotube/polydimethylsiloxane EEG electrodes was maintained by a spring-coupled pressure device. The electrodes' positions near the ears allowed to accurately track the dynamics of occipital EEG alpha rhythms. Alternatively, EEG electrodes have been integrated into caps or individualized ear pieces (9), or they may be implanted subcutaneously (10, 11) or into the skull (12–14). In all of these cases, however, the devices should be designed to be as small as possible and this is why it is imperative that they are highly energy efficient. Energy efficiency is a hallmark of biological nervous systems and a set of methods, referred to as “brain-inspired computing,” has emerged over recent years. These methods aim at replicating principles of biological nervous systems into artificial substrates and thereby allowing the design of a new generation of highly energy efficient hardware. Here, we set out to introduce computing with hyperdimensional (HD) vectors (15), one of the most powerful and elegant of these approaches.

The paper is structured as follows: In section 2, we first give an introduction to the main characteristics of computing with HD vectors. Then we provide a detailed account of how computing with HD vectors has been successfully used to detect epileptic seizures from intracranially recorded EEG signals (iEEG) in section 3. Next, we dedicate section 4 to describing examples of emerging nanotechnology hardware, which is particularly well suited for implementation of computing with HD vectors. We conclude in section 5 by summarizing our main points, giving an outlook on future developments, and important neurologic applications of computing with HD vectors. Finally, we recommend resources for further reading, mainly aimed at clinical neurophysiologists or epileptologists.

## 2. Hyperdimensional Computing

One way to conceive of computation in a very general sense is as the emergence, change, combination, and dissolution of patterns. While in biological systems, computation is considered to mainly rely on self-organization (16, 17), in the case of human artifacts, computation is engineered. In today's typical computer, the patterns used are short bit strings consisting of zeros and ones (18, 19). The central idea of computing with HD vectors, as developed by Finnish neuroscientist Pentti Kanerva, is to use random patterns that are much larger, i.e., in the order of 10,000 (15, 20). These patterns are still made from zeros and ones, but they are identically and independently distributed (i.i.d.), and are referred to as “dense random binary hypervectors” (21). The notion of a “hypervector” is due to the interpretation of these patterns as vectors or points in a very high-dimensional or “hyperspace.” Kanerva (15, 20), Plate (22), and Gayler (23) were inspired to design such a novel and unique data type of random HD vectors after studying biological central nervous systems as well as psychological models of human analogy processing. Hence, the notion of computing with HD vectors is inspired by these aspects of brains where information is often represented by very large spatiotemporal distributions of probabilistic neuronal population firing patterns (24).

**Figure 1:**
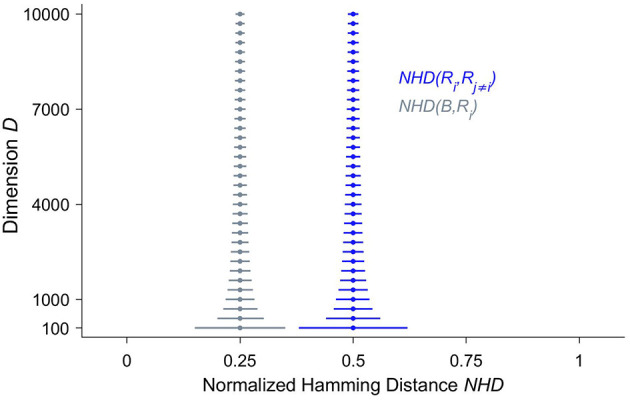
The distributions of normalized Hamming distances (*NHD*) illustrate both the non-intuitive structure of hyperdimensional space and the similarity between a bundled hyperdimensional (HD) vector and its inputs. We have selected three random dense binary HD vectors *R*_1_, *R*_2_ and *R*_3_ and bundled them together to yield the HD vector *B*, i.e., *B* = [*R*_1_ + *R*_2_ + *R*_3_]. Bundling here denotes the componentwise application of addition followed by the majority rule. Then the pairwise NHD—the number of components where two vectors are different, divided by the dimension *D*—is computed among the input vectors *NHD*(*R*_*i*_, *R*_*j*≠*i*_) shown in blue, and between *B* and each of its input vectors *NHD*(*B, R*_*i*_), displayed in gray. This procedure is repeated 50,000 times, yielding 150,000 distances for each distribution and for each dimension *D*, which increases from *D* = 100 to *D* = 10, 000 in steps of 300. The dots represent the medians, and the whiskers indicate the 1–99 percentile ranges. One can clearly observe that, with increasing dimension *D*, the normalized Hamming distances between the random vectors concentrate around 0.5. In other words, the random vectors are almost orthogonal to each other. The distances between the bundled HD vector *B* and its input vectors is much smaller and concentrates around 0.25. Both of these characteristics become progressively and smoothly—i.e., there is no sensitive dependency—more pronounced with an increasing dimension *D*.

Crucially, in HD vectors the information is equally distributed across all the bits, which are consequently all of equal importance. The HD vector as a whole and any of its parts represent the same item, though the individual parts in a less reliable manner. As a corollary, there are no most or least significant bits as in classical computing, where bits represent different values depending on their positions within the bit strings. The characteristic that the information is spread across the whole HD vector is often referred to as a holographic or holistic representation (22). The reason why holographic information representation is of fundamental importance is that it yields a very high tolerance to noise. Akin to the situation in biological brains, where very large numbers of neurons may be impaired before there are clinical manifestations (25), many bits—often in the range of 30%—of a HD vector may be randomly flipped before computing with HD vectors fails. To better understand the root causes for this surprising robustness, it is essential to study the hyperdimensional space, which the hypervectors inhabit. Given that the world we live and have evolved in can adequately be described by three spatial dimensions, at least on the scale we have direct sensory access to, it is not surprising that most of us humans do not have an intuitive grasp for hyperdimensional spaces. One such crucial but non-intuitive characteristic of hyperdimensional spaces is called “concentration of measure” (15, 26) and is illustrated in Figure 1 by the stacked blue median-and-whiskers plot. It describes the distribution of distances between dense binary HD vectors of increasing dimensionality *D*. The distance between two HD vectors is assessed by the number of components at which they differ, divided by *D*, i.e., their normalized Hamming distance. The fundamental, albeit counter-intuitive, observation is that the larger the dimension, the more the distances are concentrated around a normalized Hamming distance of 0.5. Thus, the higher the dimension, the more likely it becomes that two dense binary HD vectors differ in half of their components and are quasi-orthogonal to each other. Put differently, points in hyperdimensional space are surprisingly isolated. If one starts moving away from a point one has to traverse almost half the diameter of the hyperdimensional space until one arrives at other points, but then the number of “neighbors” starts to increase enormously. Compare this to our everyday experience of moving on a 2D plane. Kanerva points out (15), if we double the distance, the “territory” quadruples, but it will never increase billion-fold as it is possible in hyperdimensional space. Hence, in the binary hyperdimensional space, with a common but arbitrarily chosen dimension of *D* = 10,000, there exists an inconceivably large number of different binary i.i.d. HD vectors, which are quasi-orthogonal to each other. Projecting information into a hyperdimensional space therefore not only provides a massive capacity for distinct representations, but these representations are also extremely robust, because most of their neighbors are half the diameter of the whole space away. This inherent robustness is one of the main reasons that lends computing with HD vectors naturally as a computational paradigm for emerging nanoscalable hardware, where noise due to variability of the materials is a central challenge (27–31).

Moreover, it is worthwhile to mention here an interesting recent observation by Singer et al. (32) and Singer and Lazar (33) that corroborates a potential role for computing with HD vectors, or HD computing-like information processing, in biological systems as well. These authors propose that the mammalian cortex in particular might make use of HD projections of sensory information. They point out the similarities between cortical dynamics and reservoir computing (34). Here, the high-dimensional continuous activity, generated and sustained by recurrent artificial neural networks, is perturbed by localized input signals (35, 36). However, only providing a very large number of different and noise-resistant representations does not suffice to furnish an efficient computational substrate. In addition, operations are needed that allow to manipulate these representations. The two main operations for combining HD vectors are called binding and bundling. For the binary dense HD vectors, binding corresponds to the componentwise Exclusive OR function (XOR). Exclusive OR, also referred to as “exclusive disjunction,” is a logical operation that outputs “true” only, when the inputs differ. Here, we denote XOR by the circled plus sign (⊕). Importantly, the HD vector *C* that results from binding the two binary random vectors *A* ⊕ *B* is again a binary random HD vector that is quasi-orthogonal to both *A* and *B*, and hence corresponds to a new, unique, and robust combined representation. The operation XOR has four relevant characteristics. It is both commutative, *A* ⊕ *B* = *B* ⊕ *A* and associative, i.e., (*A* ⊕ *B*) ⊕ *C* = *A* ⊕ (*B* ⊕ *C*). The neutral element is 0, that is *A* ⊕ 0 = *A* and finally, XOR is self-inverse, *A* ⊕ *A* = 0. The second main operation is referred to as bundling and is basically a bitwise thresholded sum of HD vectors, yielding 1 if more than half of the components equal 1, otherwise the result is 0. In other words, bundling corresponds to a componentwise majority function. The thresholding or normalization process is essential, because it forces the resulting HD vector to remain in the binary hyperspace. Bundling of three HD vectors is denoted by [*A* + *B* + *C*], where the squared brackets represent thresholding. Note that in the case of an even number of HD vectors to be bundled, the potentially occurring draws are randomly broken. A central distinction between the binding and bundling operations is that while the HD vector resulting from binding is quasi-orthogonal to all of the HD vectors that are bound together, the HD vector yielded by bundling is similar to each of its input HD vectors, i.e., the HD vectors that are bundled together. This characteristic is demonstrated in Figure 1 by the stacked gray median-and-whiskers plot, representing the distances between the HD vectors *R*_1_, *R*_2_, *R*_3_ and their resulting bundled HD vector *B*. Therefore, bundling is well suited to represent sets of HD vectors and may function as a memory or in the case of iEEG analysis to construct “prototype” HD vectors that represent brain states of interest.

At this point, it is interesting to re-consider HD computing as being brain inspired and allowing to design cognitive architectures (37) with mechanisms and characteristics reminiscent of phenomena found in the human brain and mind. For example, the human central nervous system's essential ability to associate novel patterns (17) with already known ones might be part of the neuronal basis of metaphoric thinking, where we try to understand one abstract or not-yet-seen aspect of our world by using a different already familiar or more concrete concept (38, 39). And, on a more philosophical note, as Joseph Campbell so eloquently described in his comprehensive work (40), since the dawn of human consciousness, metaphors have been one of our most powerful tools to create coherent and meaningful stories and myths that help us navigate through our lives (41). Furthermore, one of the most promising modern concepts about consciousness is the theory of integrated information developed by Giulio Tononi et al. (42). This theory starts from the essential properties of phenomenal experience, such as the myriad of unique and different sensory impressions, which are then combined into a coherent (“integrated”) whole. These characteristics might be, at least partially, replicated within the framework of computing with HD vectors.

Before we present one way that has already been successfully used to detect epileptic seizures from EEG recorded with intracranial electrodes (iEEG), let us for the sake of completeness mention that instead of binary components (0 and 1) (21), bipolar (23), real (22), or complex (43) ones may be used to design the HD vectors. One frequent alternative choice is +1 and −1, in which case the HD vectors are termed bipolar. While these alternatives influence the type of mathematical operations used for binding and bundling and also some aspects of the hardware implementation, the overall principles and characteristics of computing with HD vectors still hold. In other words, for computing with HD vectors the high dimensionality is more important than the type of components and operations used to construct and combine the HD vectors. Furthermore, in addition to the operations of binding and bundling, HD vectors may also be permuted. Permutation is typically implemented as a circular shift of the HD vector's components and geometrically corresponds to a rotation (15). Permutation is most often used to encode sequences, for example the order of letters to classify different languages (44), or the spatiotemporal patterns of electrical muscular activity for hand gesture detection (45). Permutation might turn out to be useful in future studies to better assess the shape of iEEG signals, which has been shown to contain physiologically relevant information (46, 47).

## 3. Seizure Detection

**Figure 2:**
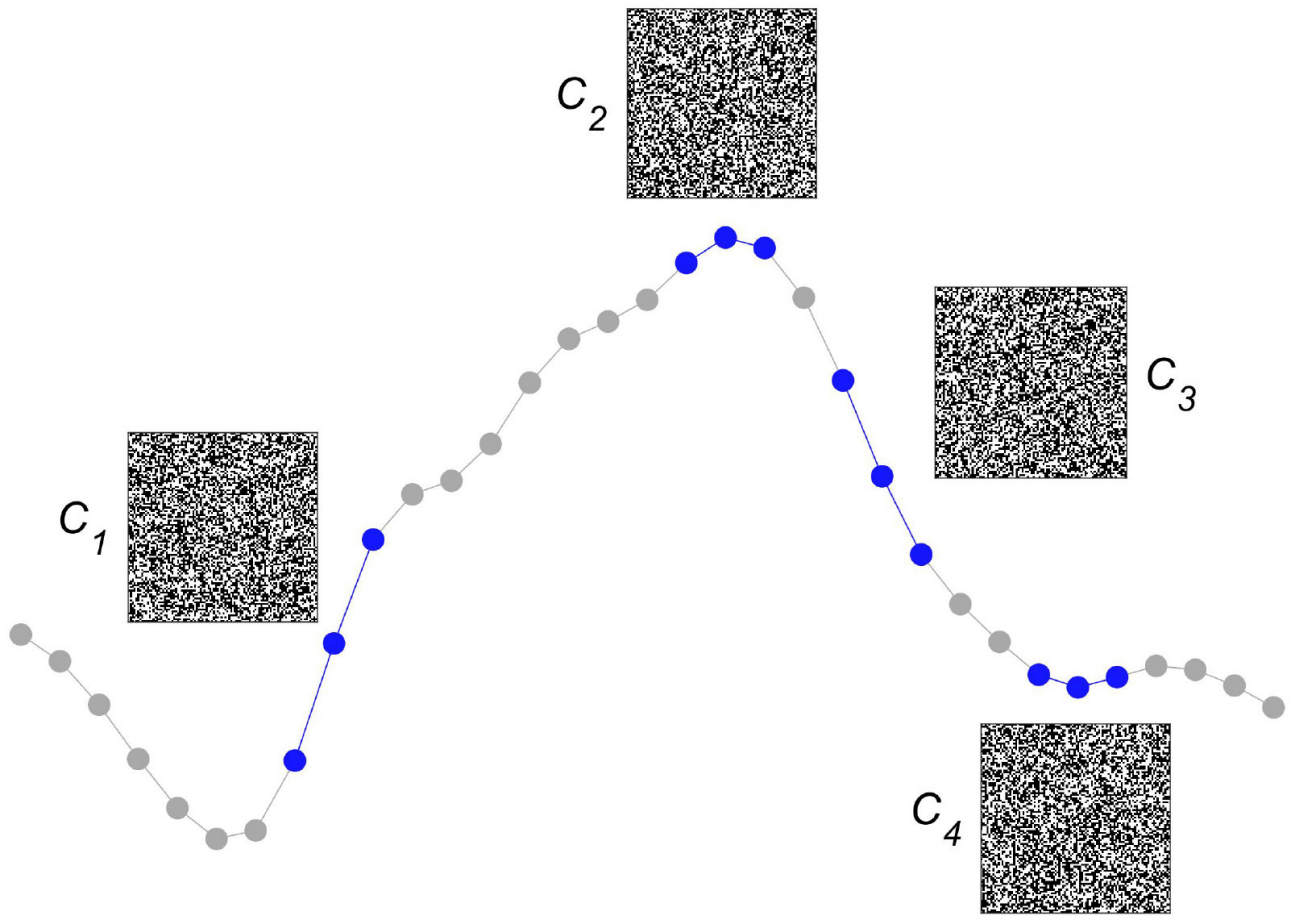
Encoding local intracranial electroencephalogram (iEEG) order relations into hyperdimensional (HD) vectors. We consider whether an iEEG sampling point is ≥ or ≱ than its preceding one. The simple situation of only three sequential iEEG sampling points, used here to demonstrate the method, yields four different possible pairs of order relations (plotted in blue), each of which is associated with a unique quasi-orthogonal binary HD vector of dimension *D* = 10, 000. For ease of graphical display, the HD vectors of size 1 x 10,000 are reshaped into squares of size 100 × 100. Each quasi-orthogonal HD vector represents a unique relation, e.g., *C*_1_ represents the relation (≥, ≥), *C*_2_ (≥, ≱),*C*_3_ (≱, ≱), and *C*_4_ (≱, ≥). These HD vectors are stored in the item memory and stay unchanged during both learning and classification.

After having explained several of the key concepts of computing with HD vectors, we now detail one way this method has been successfully used to detect epileptic seizures from iEEG. The approach starts by first assessing whether an iEEG sampling point is ≥ than its preceding one or not. Although this form of symbolization considers only order relations—or, put differently, discards all amplitude information—it has been demonstrated to be helpful for assessing smaller and larger scale iEEG dynamics (48–51). Moreover, it allows for easily building larger symbols by simply considering longer sequences of differences between sampling points. Once the iEEG has been symbolized, the next step is to create an item memory, which we choose to build dense random binary HD vectors of a dimension *D* = 10, 000. As is illustrated in Figure 2, for the didactic case of very short symbols that take only three consecutive iEEG sampling points into account, each of the four possible symbols is represented by one of the HD vectors *C*_*i*=1 : 4_. The symbols represented by quasi-orthogonal HD vectors behave like classical symbols, they are either identical or completely different. Importantly, these HD vectors, as all the elements of the item memory, will remain unchanged during both learning and classification, they represent the projections of the symbolized iEEG signals into hyperspace.

**Figure 3:**
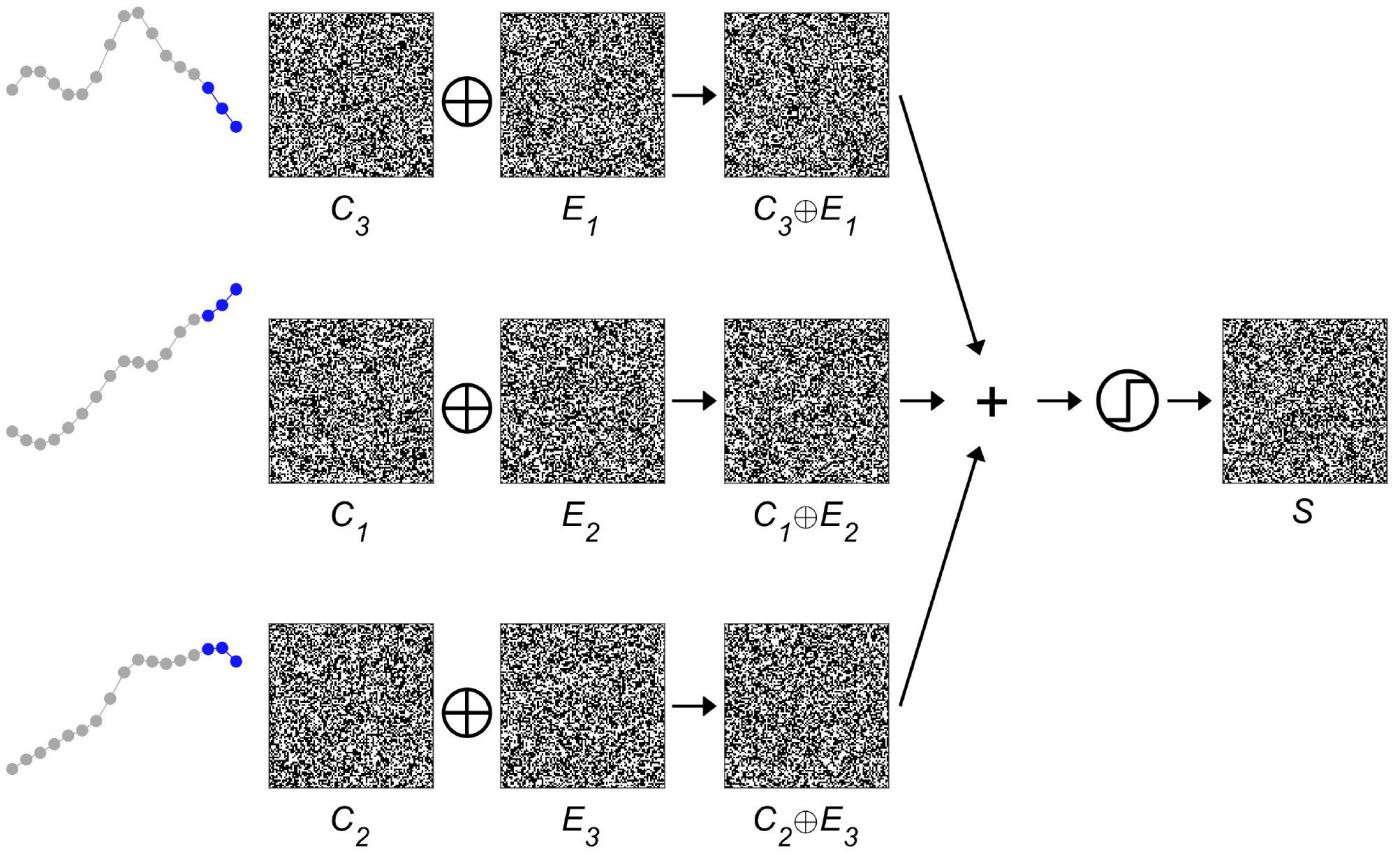
Binding and bundling of hyperdimensional (HD) vectors yield a composite representation *S* of the *spatial* intracranial electroencephalogram (iEEG) characteristics. The item memory consists of two groups of HD vectors. One group, *C*_1 : 4_, represents the four possible order relation patterns, the second group, *E*_1 : 3_, indicates the three different electrodes. By binding the corresponding pairs of *C*_*i*∈1 :4 _ and *E*_*j*∈1 : 3_, one arrives at three HD vectors that denote the type and location of the occurring order relation patterns. Bundling, that is superposing and thresholding, these three vectors produces the single composite *spatial* representation *S*, which is then further processed as a unit.

**Figure 4:**
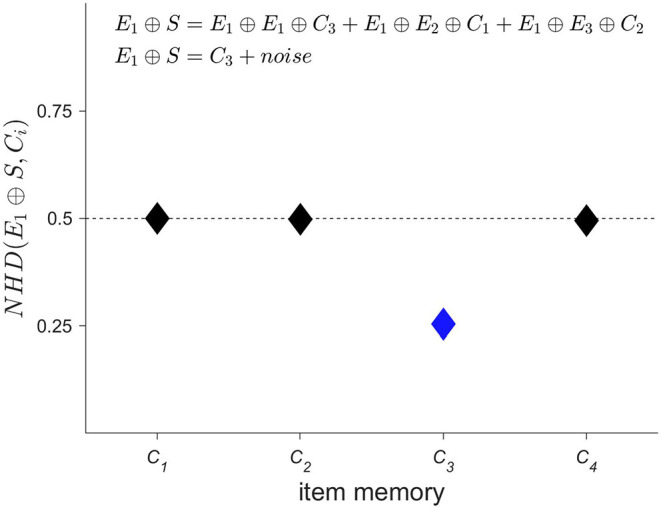
Given a spatial composite representation *S*, which order relation pattern was recorded by electrode 1? This type of question may be elegantly answered within the framework of hyperdimensional (HD) computing, by binding the HD vector representing electrode 1, i.e., *E*_1_, with the spatial composite representation *S*. Using the characteristic that XOR is its self-inverse and that binding produces new vectors that are quasi-orthogonal to their input vectors, one can interpret the result of *E*_1_ ⊕ *S* as a noisy version of *C*_3_. This noisy version is then “cleaned up” by measuring its distance to all the HD vectors of the item memory that represent order relation patterns and selecting the most similar one, i.e., *C*_3_ in the present case (displayed in blue). Comparing this result with Figure 3 shows that the correct pattern has been identified. The ability to compute robustly with approximate patterns is a hallmark of computing with HD vectors and lends it naturally as a computational paradigm for novel nano-scale memory-centric hardware with its intrinsic variability.

Next, the *spatial* information contained in the iEEG signals has to be encoded, i.e., *where* a specific-order relation pattern occurs. The corresponding procedure is displayed in Figure 3. A second set of HD vectors consisting of HD vectors *E*_*i*=1 : 3_, i.e., one per EEG electrode, is generated and added to the item memory. To compute a spatially composite representation *S* of the type and location of the occurring symbols, the HD vectors *C*_*i*_ are first bound with the HD vectors *E*_*i*_ and then bundled together, that is, superposed and thresholded. The result is a single binary HD vector of the same dimensionality as its input vectors. Thus, several HD vectors have been combined into one, which correctly implies that some information must have been lost during this process. Therefore, one refers to the resulting HD vector *S* as a *reduced* representation (43). However, despite the loss of information, computing with these approximate patterns still performs well in many practical classification tasks. The main reason for this robustness can be traced back to the counter-intuitive structure of hyperdimensional space as described in the previous section, in which HD vectors are extremely resistant toward degradation. Once again, this feature is shared with biological nervous systems, which do most often not create highly precise, but just “good enough” responses when faced with a novel challenge in our ever changing world. Interestingly, and directly related, there exist elegant ways to assess composite HD vectors such as *S*. Given a spatial composite representation *S*, one might for example wonder, which order relation pattern was recorded with electrode 1. This information may be obtained by unbinding HD vector *E*_1_ with *S*. As demonstrated in Figure 4, the result of this operation is a noisy or approximate version of *C*_3_. This noisy HD vector can be cleaned up by comparing it with the original HD vectors contained in the item memory, and using the one that has the lowest Hamming distance.

**Figure 5:**
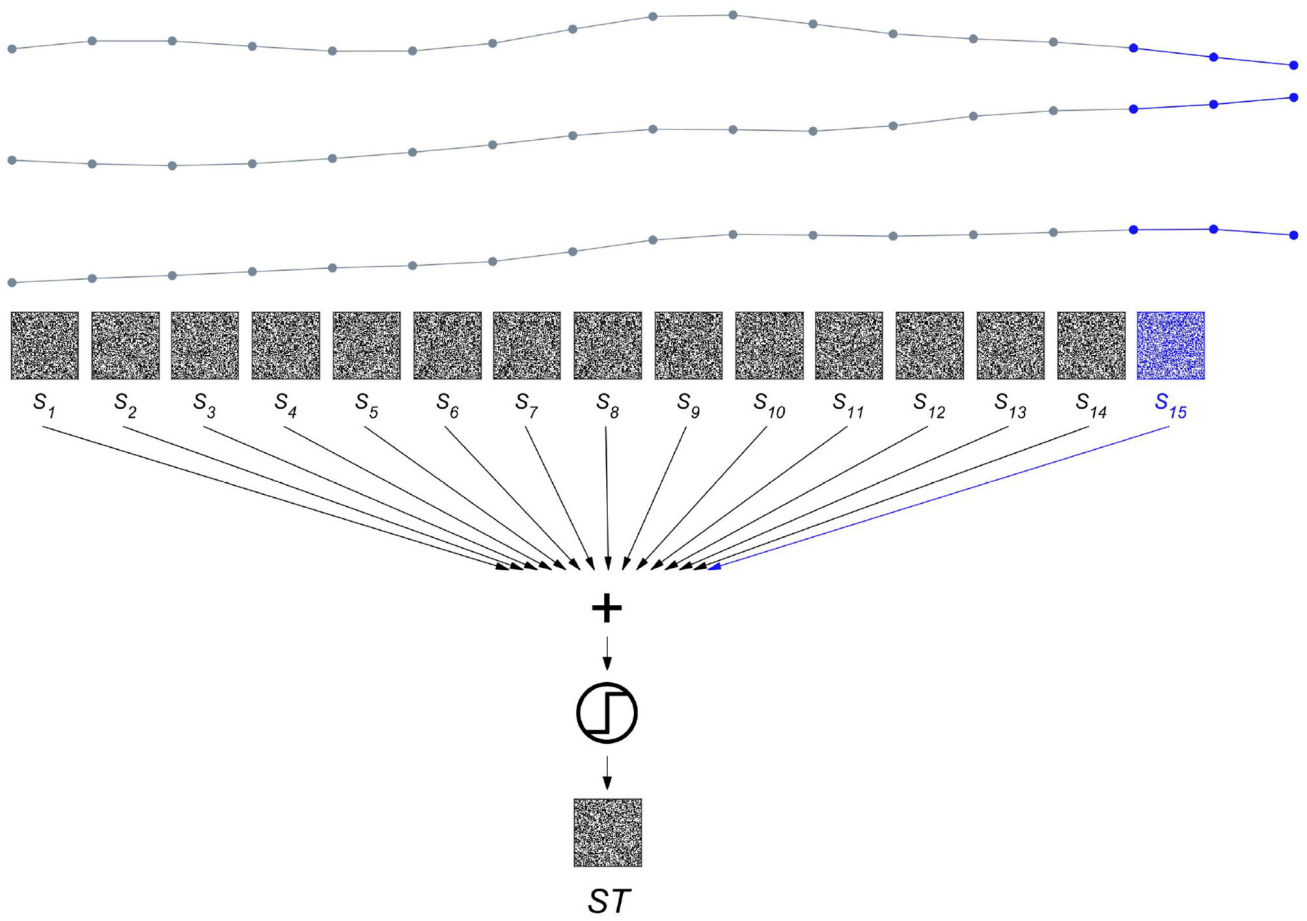
Bundling spatial representations over time produces a spatiotemporal composite representation. The three intracranial electroencephalogram (iEEG) signals are the same as shown in Figure 3, but stretched horizontally to enable the display of all the composite spatial representations *S*_*t*∈1 : 15_. *S*_15_ plotted in blue is identical to *S* of Figure 3. Each of the HD vectors *S*_*t*_ is aligned with the first of the three sampling points that are compared to yield the order relation patterns. Bundling the 15 composite representation *S*_*t*∈1 : 15_ produces the spatiotemporal composite representation *ST*.

**Figure 6:**
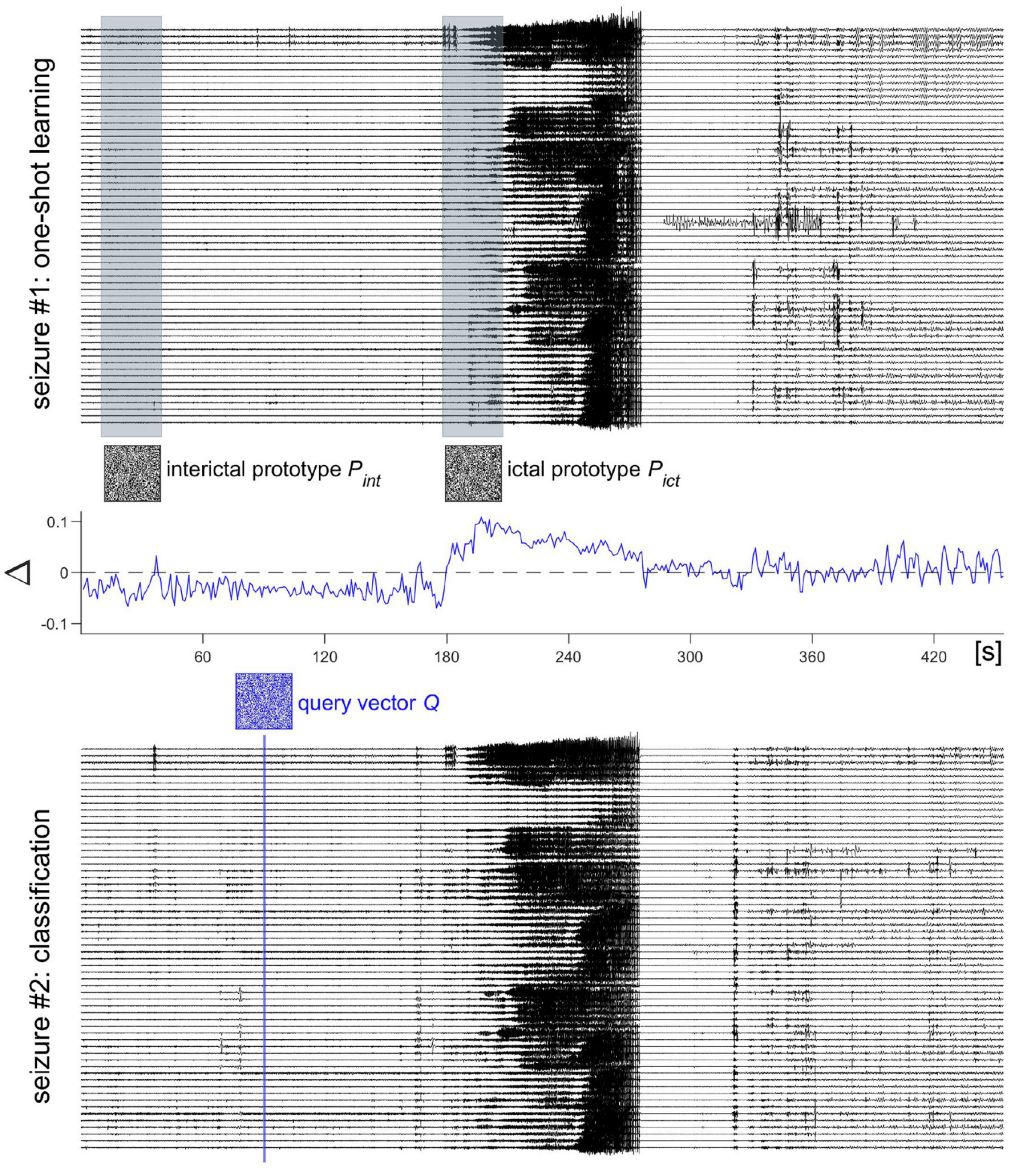
HD computing for intracranial electroencephalogram (iEEG) seizure detection. Two iEEG seizure recordings from a patient with temporal lobe epilepsy are used. The patient was implanted with intracranial electrodes with a total of 60 contacts. From seizure #1, two prototype hyperdimensional (HD) vectors are learnt, *P*_*int*_ from an interictal time period and *P*_*ict*_ from seizure onset. Both time periods have a length of 30 s and are displayed in gray. Learning consists of splitting the time periods into 30 non-overlapping moving windows of 1 s duration, computing a spatiotemporal composite representation and bundling these 30 representations into the corresponding prototypes. Seizure #2 is then used for classification. From a short moving window, again of a duration of 1 s and displayed in blue, a query HD vector *Q* is computed and its normalized Hamming distances to the interictal and ictal prototype vectors are measured. The difference Δ = *NHD*(*Q, P*_*int*_)−*NHD*(*Q, P*_*ict*_) indicates if the current brain state is closer to the ictal (Δ>0) or the interictal (Δ <0) prototype. The amplitude of Δ and its time continuously spent >0 allow defining thresholds and time periods for iEEG seizure detection with high sensitivity and specificity (50–52).

To get from a spatial to a spatiotemporal representation, the procedure shown in Figure 3 is repeated after shifting to the next sampling point in time, i.e., by using maximal overlap. The resulting set of spatial composite representations computed at timesteps *t*, *S*_*t*∈1 : 15_ are then bundled to yield a composite representation *ST* across space *and* time as displayed in Figure 5. The *ST* vectors generated from the same state are further bundled to produce a prototype HD vector representing a certain brain state. Finally, as illustrated in Figure 6, we demonstrate how this method can be applied to seizure detection. We use two iEEG recordings from a patient who suffered from pharmacoresistant temporal lobe epilepsy and underwent pre-surgical examinations at the Sleep-Wake-Epilepsy Center at the University of Bern with intracranial electrodes in order to precisely delineate the seizure onset zone. A total of 60 electrodes was used and the signals were sampled at 512 Hz and band-pass filtered between 0.5 and 120 Hz before analysis. We compared the order relations between 9 consecutive iEEG sampling points, yielding 2^8^ = 256 possible different outcomes. Therefore, in this case the item memory contained 316 random HD vectors, 60 vectors that represented the electrodes, and 256 vectors for the different order relation sequences of length 8. The recording of the first seizure is used to learn two prototype vectors *P*_*int*_ and *P*_*ict*_ that represent the interictal and the ictal brain states. Learning hereby refers to computing spatiotemporal composite representations, i.e., *ST* vectors, from two reference time periods of 30 s duration. The vectors from a reference time are bundled to produce the prototype HD vector representing the state of interest. For instance, *P*_*ict*_ = [*ST*_1_ + *ST*_2_ + …*ST*_*k*_], where all *k* HD vectors are extracted from the 30 s of the ictal state. Similarly, *P*_*int*_ is computed from the interictal reference time. It is noteworthy how simple this type of learning is. There is neither a need for a large number of seizure recordings as training examples nor for any iteration-intensive method, such as back-propagation or gradient descent. In addition, in the following classification step, a query HD vector *Q* is computed in exactly the same way as the two prototypes *P*_*int*_ and *P*_*ict*_ from the yet unseen second seizure recording. This implies that the same hardware implementation may be used for both learning and classification, another factor helpful for minimizing the energy consumption and size of an implantable device. This ability to learn from a single pass is also attractive for situations, where intermittent or continuous online learning may be needed. One might, for example, imagine situations, where the iEEG seizure patterns of a patient slightly change over time due to therapeutic interventions or a progression of the epilepsy. In that case, an update and adjustment of the ictal and interictal prototype vectors might be necessary to maintain accurate seizure detection. Coming back to the seizure detection example shown in Figure 6, the normalized Hamming distances between *Q* and both *P*_*int*_ and *P*_*ict*_ may be used to define thresholds enabling iEEG seizure detection with high sensitivity and specificity.

As indicated at the beginning of this section, symbolization based on order relation patterns combined with HD computing has recently been successfully applied to detecting epileptic seizures from short-term iEEG recordings with high sensitivity, high specificity, and high energy efficiency compared to other state-of-the-art methods (50, 52). These results have been further improved, especially in the latency of seizure detection, by including additional iEEG signal characteristics such as line length and mean amplitude (53) and, notably, could also be replicated for continuous long-term iEEG recordings (51). More specifically, these results surpass those yielded by state-of-the-art methods, including variants of deep learning (54, 55) and support vector machines (56), on the long-term dataset containing 116 seizures of 18 drug-resistant epilepsy patients in 2,656 h of recordings. HD computing trains 18 patient-specific models by using only 24 seizures: 12 models are trained with one seizure per patient, the others with two seizures. The trained models detect 79 out of 92 unseen seizures without false alarms. Importantly, a simple implementation of HD computing on the contemporary Nvidia Tegra X2 embedded device achieves 1.7× –3.9× faster execution and 1.4× –2.9× lower energy consumption compared to the best result from the state-of-the-art methods [see (51) for more details].

We conclude this section by mentioning that, while we have described the use of intra-cranially recorded electric brain signals for HD computing based seizure detection here, it has recently been shown that the method can also be successfully applied to extra-cranially recorded surface EEG signals, which typically contain more movement and muscular artifacts than iEEG (57). In the next section, we focus on viable hardware implementations of HD computing. Particularly, we go beyond the contemporary hardware fabric where HD computing has already shown energy efficiency benefits compared to the other approaches. We provide an overview on how HD computing can benefit from such emerging fabrics.

## 4. Analog In-Memory Computing Hardware

We have seen that computing with HD vectors allows to construct highly efficient algorithms, mainly because training is very fast and only uses relatively few steps compared to other approaches such as deep learning, which depends on many iterations to adjust the parameters of its artificial neurons. This single pass, non-iterative type of training is akin to our own neuro-cognitive ability to learn, to keep continuously learning, and to not forget certain events, the latter typically associated with the experience of intense emotions. However, for practical purposes, it is central that not only the algorithms but also the physical substrates into which the algorithms are implemented are efficient, i.e., allow for minimizing energy consumption. Only combining energy efficient algorithms with energy efficient hardware will ultimately enable the design of extremely small devices that can be implanted into the human brain in a minimally invasive way (58, 59), and—once again—biological brains provide inspiration for potential solutions.

John von Neumann, the ingenious Hungarian-American mathematician, physicist and polymath, who among many other achievements also designed the architecture that is still prevalent in most of today's computers (and which are accordingly called von Neumann architectures in his honor), already pointed out some of the major distinctions between biological brains and engineered information processing devices. In his last published and impressively visionary book titled “The Computer and the Brain” (60), he underlines that, although the building elements of biological brains, or “natural automata” as he calls them, are much slower and much less precise than their artificial counterparts, they are significantly more energy efficient, work in parallel, and are much more tightly arranged. The latter observation has inspired modern strategies to design non-von Neumann architectures by co-locating memory and computation. As a result, in the non-von Neuman architectures, computations are local and the global interconnects are accessed at a relatively low frequency, as is a hallmark of biological brains molded through evolution, where efficient structures with short communication distances had a selective advantage (61, 62).

**Figure 7:**
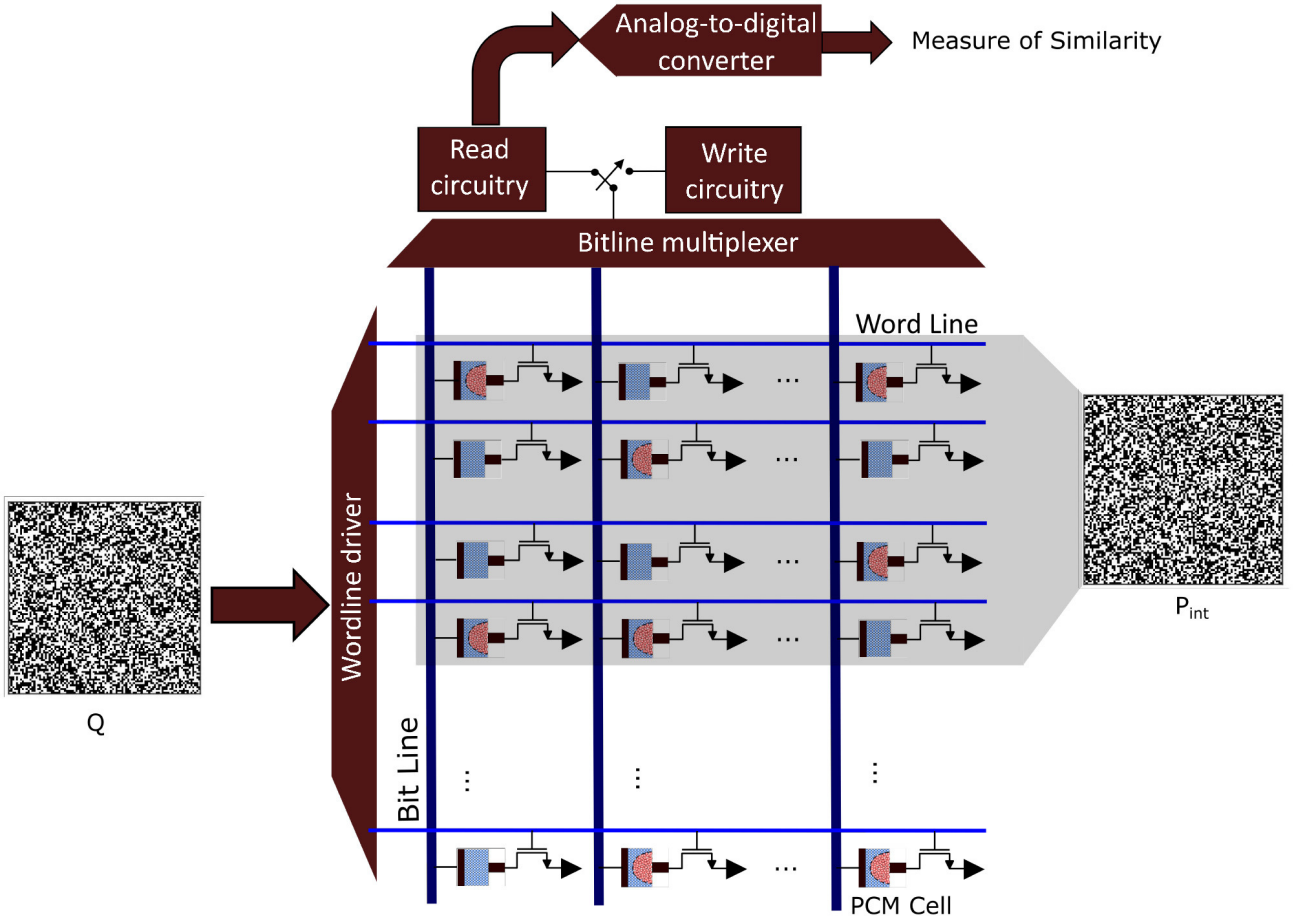
Analog in-memory computing hardware based on phase change material (PCM) devices that are arranged into a crossbar array. In a programming phase, each binary prototype hyperdimensional (HD) vector (*P*_*int*_ or *P*_*ict*_) is written into 10,000 PCM cells. This programming is done by applying a write voltage that changes the conductance state of the PCM cells according to the corresponding binary HD vector component. For classification, the query binary HD vector (*Q*) is applied to the crossbar array, and its similarity is measured with the programmed prototypes.

In classical von Neumann architectures, data have to be shuffled from memory to the central arithmetic logic unit and back, whereas in brains, information processing and at least some forms of memory formation take place within the same structures, such as the synapses that connect neocortical neurons. Synapses change their electrical resistance depending on their electrical activity, a characteristic that has been replicated in analog memristive devices, such as resistive random access memory (RRAM) and phase-change material (PCM) devices, leading to in-memory computing hardware [see (63) for an overview]. All these emerging nanotechnologies are characterized by a high variability of their components, and they thus rely on computational paradigms that not only compensate but may even embrace randomness. Importantly, it has recently been demonstrated that HD computing can be implemented on large-scale PCM devices arranged into crossbar arrays. It maintained a very high accuracy for various classification tasks with excellent energy efficiency (30). The used HD computing architecture is similar to the one described here for the iEEG seizure detection. Figure 7 illustrates how the interictal and ictal prototypes can be mapped onto a PCM crossbar array where the *Q* HD vector is applied for classification. Interestingly, HD computing was used to compensate the intrinsic variabilities of the nanoscale devices on the one hand, but on the other hand these very variabilities were exploited to optimize HD projections (29, 31). This is akin to an intriguing idea put forward in the context of interpreting cortical dynamics as a biological realization of reservoir computing, namely that the variability, diversity, and randomness of neurons may also promote HD projections (36). Furthermore, the aforementioned prototypes (29, 31) achieve shorter communication pathways by using 3D monolithic integration, instead of only 2D, to construct a layered design, similar for example to the neocortex of human brains. To summarize and conclude this section, many of these emerging nano-materials are inherently stochastic and need a computational paradigm that is robust and therein lies the great appeal to invoke computing with HD vectors for future miniaturized and thus much better implantable iEEG monitoring systems.

## 5. Conclusions and Outlook

We have presented the key concepts of HD computing and how the method has been successfully used for detecting epileptic seizures from iEEG with high energy efficiency, high specificity, and high sensitivity. We have described how HD computing relies on bit-wise operations, is highly parallel, memory-centric, scalable, and robust thanks to the counterintuitive structure of hyperdimensional space, where points are relatively isolated. Furthermore, computing with HD vectors is transparent and explainable, in the sense that each step during learning or classification is easily understandable. This contrasts to other methods used in artificial intelligence, which are often likened to “black boxes,” where even the designers are not able to comprehend the details of why a system arrived at a particular solution. Considering all these characteristics, we regard HD computing as a flexible paradigm ideally suited for a new generation of implantable devices for monitoring electrical brain activity, which will be significantly more energy efficient and will consequently hopefully usher in personalized epileptology on a previously unknown scale.

There are many potential future clinical applications for HD computing based (i)EEG analysis. One compelling recent insight, for example, is how tightly and probably causally connected neurodegeneration is with both the impairment of slow wave sleep (64, 65) and epileptic activity (66–68). Both—slow wave sleep and epileptic activity—might in the future be monitored for ultra long-term time periods, i.e., for months to years, at the patient's home with robust and easy to use (i)EEG devices that make use of computing with HD vectors for a smaller and therefore less obtrusive design. From a neurologic point of view, tracking both sleep and epileptic activity might turn out to be crucial, for the latter might be exacerbated by improving slow wave sleep. This might potentially necessitate the use of drugs or a combination of drugs that simultaneously stabilize sleep and suppress or minimize epileptic activity, such as trazodone (69) together with levetiracetam (70, 71). Ultra long-term monitoring might also provide essential information about whether interictal epileptic activity has a similarly aggravating effect on neurodegeneration as does seizure activity. Such insights are essential for optimizing treatments with drugs like gabapentin that have been reported to improve slow wave sleep (72) and suppress seizures (73), but increase interictal activity (74).

Another very interesting recent development in epileptology is the growing understanding of how brain–body interactions (75) might give rise to epileptic seizures and epilepsy. In particular, widespread cardiovascular disorders such as arterial hypertension may be associated with or even cause epileptogenic effects (76, 77). Therefore, the multi-modal integration of EEG and non-EEG digital biomarkers (58) is a highly promising approach to monitoring the mutual interactions between the visceral organs and the nervous system. For such multi-modal monitoring, HD computing lends itself naturally as a highly efficient computational paradigm for sensory fusion (78). This type of multi-sensor monitoring may be considered a specific example of “digital phenotyping,” a concept recently introduced by Onnela et al. (79) as the moment-by-moment quantification of the individual-level human phenotype *in situ* using data from smartphones and other personal digital devices. Though the main field of investigation for Onnela is psychiatry, digital phenotyping of patients suffering from neurological disorders might inform personalized care as well.

On a more technical side, a very active area of research are memory-augmented neural networks (MANNs), in which a deep neural network is connected to an associative memory for fast and lifelong learning. Computing with HD vectors can reduce the complexity of MANNs by computing with binary vectors (80). This recently proposed method reduced the number of parameters by replacing the fully connected layer of a convolutional neural network with a binary associative memory for EEG-based motor imagery brain–machine interfaces (81). Other interesting developments are the combination of concepts from HD and reservoir computing, which uses recurrent connections in a neural network to create a complex dynamical system (35, 82). Kleyko et al. (83) demonstrated the similarities between the design and manipulation of HD binary vectors and the random projections of input values onto a binary reservoir, its updating, and its nonlinearity. Another recent innovative approach is to combine HD computing with event-driven inputs such as dynamic vision sensors (84). Hersche et al. (85) showed how to embed features extracted from such spiking sensors into binary sparse representations to reduce the complexity of downstream tasks such as regression.

We conclude by providing some recommendations for further reading, in particular intended for clinical neurophysiologists and epileptologists who want to learn more about the mathematical and engineering aspects of computing with HD vectors. In our opinion, the best way to delve deeper into this captivating field is by reading the seminal paper by Pentti Kanerva (15), followed by Tony Plate's book (43). An introduction to computing with HD vectors for robotics, which also includes instructive visualizations to further a more intuitive understanding of hyperdimensional spaces, has been written by Neubert et al. (86). Furthermore, there are two important books that are highly relevant for the use of HD computing to better understand and model biological brains (37) on the one hand, and describe in detail recent and potential future implementations in new types of hardware (87) on the other hand. We hope that this literature will provide a helpful entry point into the fascinating world of HD computing and will thereby promote its medical applications to ultimately improve the care for epilepsy patients.

## Data Availability Statement

Publicly available datasets were analyzed in this study. This data can be found here: http://ieeg-swez.ethz.ch/.

## Author Contributions

KS and AR: conception, writing of manuscript, and design of figures. KS: iEEG data acquisition and analysis, Matlab Code for Figures 1–6. Both authors significantly contributed to the article and approved the submitted version.

## Conflict of Interest

AR was employed by the company IBM (Switzerland). The remaining author declares that the research was conducted in the absence of any commercial or financial relationships that could be construed as a potential conflict of interest.
